# mRNA booster immunization elicits potent neutralizing serum activity against the SARS-CoV-2 Omicron variant

**DOI:** 10.1038/s41591-021-01676-0

**Published:** 2022-01-19

**Authors:** Henning Gruell, Kanika Vanshylla, Pinkus Tober-Lau, David Hillus, Philipp Schommers, Clara Lehmann, Florian Kurth, Leif E. Sander, Florian Klein

**Affiliations:** 1 Laboratory of Experimental Immunology, Institute of Virology, Faculty of Medicine and University Hospital Cologne, University of Cologne, Cologne, Germany; 2 Department of Infectious Diseases and Respiratory Medicine, Charité – Universitätsmedizin Berlin, Freie Universität Berlin and Humboldt-Universität zu Berlin, Berlin, Germany; 3 Department I of Internal Medicine, Faculty of Medicine and University Hospital Cologne, University of Cologne, Cologne, Germany; 4 German Center for Infection Research, Partner site Bonn-Cologne, Cologne, Germany; 5 Center for Molecular Medicine Cologne (CMMC), University of Cologne, Cologne, Germany; 6 Department of Tropical Medicine, Bernhard Nocht Institute for Tropical Medicine and Department of Medicine I, University Medical Center Hamburg-Eppendorf, Hamburg, Germany

**Keywords:** Antibodies, Vaccines, SARS-CoV-2

## Abstract

The Omicron variant of SARS-CoV-2 is causing a rapid increase in infections across the globe. This new variant of concern carries an unusually high number of mutations in key epitopes of neutralizing antibodies on the viral spike glycoprotein, suggesting potential immune evasion. Here we assessed serum neutralizing capacity in longitudinal cohorts of vaccinated and convalescent individuals, as well as monoclonal antibody activity against Omicron using pseudovirus neutralization assays. We report a near-complete lack of neutralizing activity against Omicron in polyclonal sera from individuals vaccinated with two doses of the BNT162b2 COVID-19 vaccine and from convalescent individuals, as well as resistance to different monoclonal antibodies in clinical use. However, mRNA booster immunizations in vaccinated and convalescent individuals resulted in a significant increase of serum neutralizing activity against Omicron. This study demonstrates that booster immunizations can critically improve the humoral immune response against the Omicron variant.

## Abstract (web summary)

Neutralization of the SARS-CoV-2 Omicron variant is markedly impaired in sera from recipients of two doses of the COVID-19 vaccine BNT162b2 or from convalescent individuals, but is robustly increased in both groups following a booster vaccine dose.

## Main

Most approved coronavirus disease 2019 (COVID-19) vaccines are based on transient expression of the viral spike (S) glycoprotein (derived from the Wu01 strain) to induce severe acute respiratory syndrome coronavirus 2 (SARS-CoV-2)-directed immunity^1^. Mutations in antibody epitopes on the spike protein can result in increased viral resistance to neutralizing antibodies and have been associated with reduced vaccine effectiveness^2^. Moreover, they are able to strongly impair the activity of monoclonal antibodies used for the treatment and prevention of COVID-19 (ref. ^3^). Emerging viral variants that escape the antibody response can therefore threaten the success of critical measures against SARS-CoV-2 infection.

Shortly after its identification in the Gauteng province in South Africa, the Omicron variant of SARS-CoV-2 (BA.1 sublineage of B.1.1.529) was designated as a variant of concern (VOC) by the World Health Organization (WHO). Genomic surveillance and surrogate parameters (for example, S gene target failure in diagnostic polymerase chain reaction tests) document a sharp rise in Omicron infections across the globe^4^. Moreover, the observation of increasing incidence in populations with a high prevalence of SARS-CoV-2 immunity as well as reports of re-infection suggest that the Omicron variant possesses potent immune evasion properties^5^. Compared with previously described VOCs, the Omicron variant is notable for its high number of non-synonymous mutations relative to the ancestral Wu01 strain of SARS-CoV-2. The majority of these mutations are located in the viral spike glycoprotein and include critical epitopes for SARS-CoV-2-neutralizing antibodies in the N-terminal domain and the receptor-binding domain (RBD). Several of these mutations have already been associated with resistance to SARS-CoV-2- or vaccine-induced neutralizing antibodies^3,6,7^. Therefore, the spread of the Omicron variant can have important implications for current strategies to prevent and treat COVID-19, and may require urgent public health interventions to limit transmission and morbidity.

**Fig. 1:**
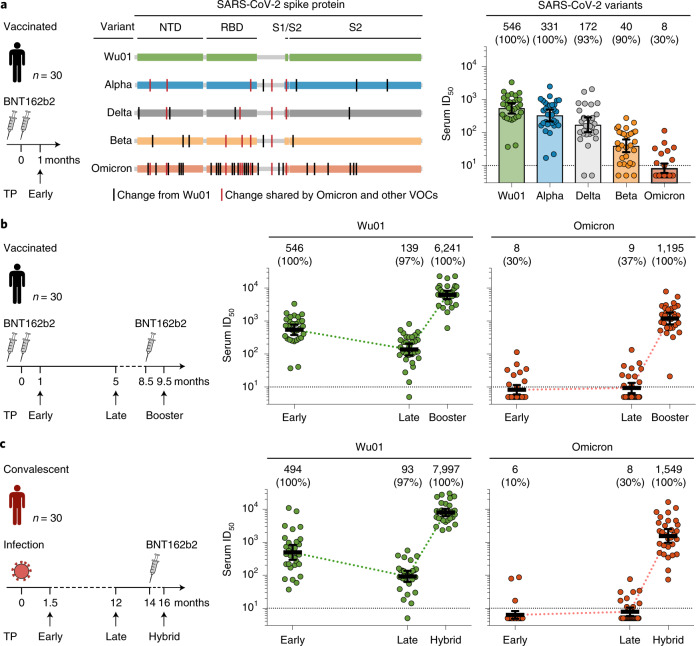
SARS-CoV-2-neutralizing serum activity in vaccinated and convalescent individuals. **a**, Neutralizing serum activity was determined in samples obtained 1 month after two doses of BNT162b2 against the ancestral Wu01 strain of SARS-CoV-2 and four VOCs. Residues with changes relative to the Wu01 strain are indicated by intersecting lines. Fifty percent inhibitory serum dilutions (ID_50_s) were determined by pseudovirus neutralization assays. Bars indicate geometric mean ID_50_s with 95% confidence intervals (CIs). Numbers above the graph indicate the geometric mean ID_50_﻿s, and the percentages of samples with detectable neutralizing activity above the lower limit of quantification (LLOQ) are given in parentheses. **b**, Serum ID_50_s against the Wu01 strain and the Omicron variant of SARS-CoV-2 in a longitudinal cohort of 30 vaccinated individuals. Samples were collected at a median of 1 month (Early) and 5 months (Late) after two doses of BNT162b2, and 1 month after a subsequent single dose of BNT162b2 (Booster). Colored lines connect geometric mean ID_50_s and error bars indicate the 95% CIs. **c**, Serum ID_50_s against the Wu01 strain and the Omicron variant of SARS-CoV-2 in a longitudinal cohort of 30 COVID-19-convalescent individuals. Samples were collected at a median of 1.5 months (Early) and 12 months (Late) after diagnosis of SARS-CoV-2 infection, as well as 1.5 months after a single BNT162b2 vaccination (Hybrid). Colored lines connect geometric mean ID_50_s and error bars indicate 95% CIs. In **a**, **b** and **c**, ID_50_s below the LLOQ (ID_50_ of 10, indicated by the black dashed lines) were imputed to half the LLOQ (ID_50_ = 5). NTD, N-terminal domain; TP, time point.

To determine the susceptibility of the Omicron variant to vaccine-induced serum activity, we analyzed samples obtained from 30 individuals with no evidence of prior infection^8^. Samples were collected 1 month (median, 4 weeks; range, 3–6 weeks; ‘Early’ time point) after completion of a two-dose course of the BNT162b2 vaccine (Fig. 1a). Study participants had a median age of 49 years (range, 27–78 years) and a nearly equal sex distribution (57% female participants, 43% male participants; Supplementary Table 1). Neutralizing activity was determined using an established lentivirus-based pseudovirus assay. Results obtained in pseudovirus assays typically correlate well with those obtained against authentic virus^9^. Sera were tested against pseudoviruses expressing the spike proteins of the Wu01 vaccine strain, or of the Alpha (B.1.1.7), Delta (B.1.617.2), Beta (B.1.351), or Omicron VOCs (Fig. 1a and Supplementary Table 2). All samples showed neutralizing activity against the Wu01 strain with a geometric mean 50% inhibitory serum dilution (GeoMean ID_50_) of 546 (Fig. 1a). Serum neutralizing activity against the Alpha, Delta, and Beta variant was decreased to GeoMean ID_50_s of 331, 172, and 40, respectively (samples that did not achieve 50% inhibition at the lowest tested dilution of 10 were imputed to an ID_50_ of 5). Notably, only nine out of the 30 vaccinated individuals (30%) had detectable serum neutralizing activity against Omicron, resulting in a GeoMean ID_50_ of 8 (Fig. 1a), which was significantly lower than against the Beta variant (*P* < 0.0001), one of the most immune evasive variants previously described^2^.

To investigate the change in serum neutralizing activity against the Omicron variant over time and assess the impact of a booster vaccination, we analyzed longitudinal samples of the 30 vaccinees. Neutralizing serum activity against Wu01 and Omicron was determined at 5 months (median, 21 weeks; range, 19–31 weeks; ‘Late’ time point) after the second BNT162b2 vaccination, as well as at 1 month (median, 3 weeks; range, 2–12 weeks) after a single BNT162b2 booster dose (median, 41 weeks after the second vaccination; range, 30–44 weeks; ‘Booster’ time point; Fig. 1b). After completion of the two-dose course of the BNT162b2 vaccine, neutralizing activity against Wu01 decreased fourfold during the period of 5 months from a GeoMean ID_50_ of 546 to 139, but was strongly increased after booster vaccination (GeoMean ID_50_ of 6,241). Serum neutralizing activity against the Omicron variant after two vaccine doses remained low, with only 30–37% of the samples showing detectable neutralization, resulting in GeoMean ID_50_s of 8 and 9 at the Early and Late time points, respectively. However, neutralizing serum activity to the Omicron variant increased by more than 100-fold after the booster dose of BNT162b2, resulting in a GeoMean ID_50_ of 1,195, and was detectable in all 30 participants (100%) (Fig. 1b). Notably, serum neutralizing activity against the Omicron variant following booster immunization was even higher than neutralizing titers against Wu01 after two doses of BNT162b2 (GeoMean ID_50_ of 1,195 versus 546; *P* = 0.0003).

We also analyzed the neutralizing serum response to the Omicron variant in a longitudinal cohort of 30 previously unvaccinated COVID-19 convalescent individuals (Supplementary Table 1)^10^. Study participants were followed for up to 16 months from the time of SARS-CoV-2 infection, which occurred between February and March 2020 (that is, prior to the emergence of WHO-designated VOCs). Serum samples were collected at 1.5 months (range, 4–10 weeks; Early time point) and at 12 months (range, 49–59 weeks; Late time point) after the diagnosis of infection (Fig. 1c). In addition, serum was obtained 1 month (median, 6 weeks; range, 4–10 weeks) after a single dose of BNT162b2, resulting in a hybrid immunity acquired from a combination of infection and vaccination (median, 67 weeks; range, 62–71 weeks from disease onset; ‘Hybrid’ time point; Fig. 1c). Early after infection, neutralizing activity against Wu01 was variable, ranging in ID_50_s from 37 to 11,008, with a GeoMean ID_50_ of 494 that decreased to 93 after 12 months (Late). Following a single BNT162b2 dose, all convalescent individuals had a strong increase in neutralizing serum activity resulting in a GeoMean ID_50_ of 7,997 against Wu01 (Hybrid). No neutralization (that is, no detectable ID_50_) or only weak neutralizing activity was detected against the Omicron variant in samples collected from convalescent individuals at the Early and Late time points. However, a slight increase in neutralizing activity was observed at the Late time point in some individuals, potentially indicating ongoing affinity maturation resulting in antibodies capable of targeting a wider spectrum of SARS-CoV-2 variants (Supplementary Table 2). Despite the near-lack of neutralizing serum activity against the Omicron variant at a median of 12 months after SARS-CoV-2 infection (GeoMean ID_50_ of 8; Late time point), a single dose of BNT162b2 induced a strong increase, demonstrated by a GeoMean ID_50_ of 1,549 1 month after vaccination (Hybrid time point) (Fig. 1c). We conclude that the Omicron variant exerts substantial humoral immune escape in BNT162b2-vaccinated and convalescent individuals. However, high levels of neutralizing activity against the Omicron variant can be induced by a BNT162b2 booster immunization.

**Fig. 2:**
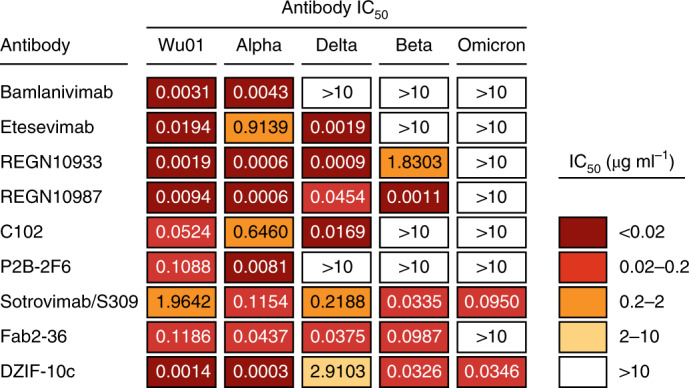
SARS-CoV-2-neutralizing activity of monoclonal antibodies. Fifty percent inhibitory concentrations (IC_50_s) of monoclonal antibodies against the ancestral Wu01 strain of SARS-CoV-2 and four variants of concern determined by pseudovirus neutralization assays. IC_50_ > 10 µg ml^−1^ indicates failure to achieve 50% neutralizing activity at the highest tested antibody concentration of 10 µg ml^−1^.

Finally, we investigated whether the high number of mutations in the Omicron variant affects the activity of SARS-CoV-2-neutralizing monoclonal antibodies, which have been demonstrated to effectively reduce COVID-19-associated morbidity and mortality^11,12^. To this end, we assessed the activity of nine monoclonal antibodies, encompassing antibodies authorized for clinical use (bamlanvimab, etesevimab, REGN10933 (casirivimab), REGN10987 (imdevimab) and S309 (sotrovimab)), an antibody in clinical investigation (DZIF-10c), and additional antibodies representative of different classes of antibodies targeting the RBD of the SARS-CoV-2 spike protein (P2B-2F6, C102 and Fab2-36) (Fig. 2). All antibodies were tested in parallel in the pseudovirus neutralization assay against the Wu01 strain as well as the Alpha, Delta, Beta, and Omicron variants. Although all antibodies showed neutralizing activity against Wu01 and Alpha, only seven out of nine and five out of nine showed neutralizing activity against the Delta and Beta variants, respectively (Fig. 2). Notably, neutralizing activity against the Omicron variant was abolished in seven out of nine antibodies (Fig. 2). We conclude that the neutralizing activity of several monoclonal antibodies is markedly reduced against the Omicron variant and may limit treatment options for Omicron-induced COVID-19.

The rapid surge in Omicron variant infections poses a significant challenge to public health. This study demonstrates the marked resistance of the Omicron variant to serum neutralizing activity induced by two doses of the BNT162b2 vaccine or SARS-CoV-2 infection. Lower neutralizing titers have been associated with an increased risk of symptomatic COVID-19, suggesting that limited neutralizing activity against Omicron may result in increased risk of infection and higher burden of disease^13^. Whether cellular immunity will be effective in preventing severe disease after Omicron infection in the absence of a potent neutralizing antibody response remains to be determined^14,15^. Importantly, a single BNT162b2 booster immunization effectively induced a substantial increase in serum neutralization against the Omicron variant and resulted in neutralizing titers similar to those observed against Wu01 after two doses of BNT162b2. This analysis was limited to mostly healthy individuals receiving the BNT162b2 vaccine. However, the overall comparable immunogenicity of mRNA-1273 suggests that booster immunizations with both approved mRNA vaccines may yield similar results^16^. We speculate that the extent of the potent neutralizing response against the Omicron variant following booster vaccination may be a consequence of ongoing affinity maturation following initial vaccination or SARS-CoV-2 infection with the ancestral spike protein^17–19^.

There is a critical need to make vaccines globally available to counter the emergence of novel variants. Although variant-specific vaccines and novel monoclonal antibodies may be required for optimal activity, the present data provide evidence that booster immunization with BNT162b2 can induce robust neutralization against the immune-evasive Omicron variant. However, higher levels of neutralizing serum activity might not necessarily prevent SARS-CoV-2 infection, as indicated by Omicron infections in boostered individuals. Finally, follow-up analyses will be required to determine the durability of the neutralizing antibody response to Omicron after booster immunization^20^.

## Methods

### Study design

Samples from COVID-19-convalescent individuals were obtained under protocols approved by the ethics committee of the Medical Faculty of the University of Cologne (16–054 and 20–1187). Individuals with a history of polymerase chain reaction (PCR)-confirmed SARS-CoV-2 infection (as reported by the participants to investigators, either verbally or by providing a positive test certificate) were enrolled at the University Hospital Cologne (Germany) between April and May 2020, ≤10 weeks after diagnosis and/or symptom onset, and followed longitudinally. Except for one participant who was hospitalized during acute SARS-CoV-2 infection, all individuals reported having mild SARS-CoV-2 infection-related symptoms. Although written proof of positive PCR testing was not formally required for study participation, the detectable SARS-CoV-2-neutralizing activity in all participants at baseline (prior to the availability of COVID-19 vaccines) provided independent confirmation of previous SARS-CoV-2 infection. Participants in the convalescent cohort were followed longitudinally to evaluate the long-term immune response to SARS-CoV-2, and booster immunizations (that is, vaccinations in the previously unvaccinated convalescent individuals) were performed as part of routine care. Samples for analysis in this study were identified based on information provided by the participants during regular follow-up visits with regard to whether and which COVID-19 vaccine (that is, booster immunization) they had received at what time.

Samples from non-infected vaccinated individuals were obtained under protocols (EICOV, COVIMMUNIZE and COVIM) approved by the ethics committee of Charité – Universitätsmedizin Berlin (EA4/245/20 and EA4/244/20; EICOV and COVIMMUNIZE), and by the Federal Institute for Vaccines and Biomedicines (Paul Ehrlich Institute) and the ethics committee of the state of Berlin (COVIM). Participants in the observational vaccinated cohort studies were followed longitudinally to evaluate the immune response to approved SARS-CoV-2 vaccinations. Participation was offered to health-care workers vaccinated at the Charité – Universitätsmedizin (Berlin, Germany) as well as to elderly individuals (≥70 years) at a general practice (Berlin, Germany) irrespective of medical condition. Initial vaccinations as well as booster immunizations were independently performed as part of routine care outside of the cohort study. Samples for analysis in this study were selected post-hoc (that is, after booster immunizations were performed). All participants underwent nucleic acid amplification testing for SARS-CoV-2 at the time of sampling, and all samples were tested for the presence of anti-nucleocapsid antibodies using the SeraSpot Anti-SARS-CoV-2 IgG microarray-based immunoassay (Seramun Diagnostica). Samples from individuals with a history of SARS-CoV-2 infection (as reported by participants to investigators) or confirmed infection (positive nucleic acid amplification test or anti-nucleocapsid antibodies) were not included for analysis of the vaccinated cohort in this study.

All participants in the convalescent and vaccinated cohorts provided written informed consent. Selection of samples and participants for this study was based on receipt of the identical vaccine (BNT162b2), on the availability of largely comparable sampling time points (before and after immunizations), on a comparable and broad range of individuals of different age, and on a comparable and balanced sex distribution. Summarized details on the participants analyzed in this study are listed in Supplementary Table 1.

### Cloning of SARS-CoV-2 spike constructs

The Omicron (BA.1 sublineage of B.1.1.529) spike construct was based on amino acid substitutions observed in initial isolates (EPI_ISL_6640916, EPI_ISL_6640917, EPI_ISL_6640919) and it carries the following changes in the spike protein compared with the Wu01 strain (EPI_ISL_406716): A67V, Δ69–70, T95I, G142D, Δ143–145, Δ211, L212I, ins214EPE, G339D, S371L, S373P, S375F, K417N, N440K, G446S, S477N, T478K, E484A, Q493R, G496S, Q498R, N501Y, Y505H, T547K, D614G, H655Y, N679K, P681H, N764K, D796Y, N856K, Q954H, N969K and L981F. Codon-optimized overlapping gene fragments (Thermo Fisher) were assembled and cloned into the pCDNA3.1/V5-HisTOPO vector (Thermo Fisher, cat. no. K480001) using the NEBuilder HiFi DNA Assembly Kit (New England Biolabs, cat. no. E5520S). The Alpha (B.1.1.7, with Δ69–70, Δ144, N501Y, A570D, D614G, P681H, T716I, S982A and D1118H changes), Beta (B.1.351, with D80A, D215G, Δ242–244, K417N, E484K, N501Y, D614G and A701V changes), and Delta (B.1.617.2, with T19R, G142D, Δ156–7, R158G, L452R, T478K, D614G, P681R and D950N changes) spike variants were produced by PCR-induced mutagenesis, and the assembly of PCR products was performed using the NEBuilder HiFi DNA Assembly Kit. All plasmid sequences were confirmed by sequencing.

### Monoclonal antibodies

Antibodies S309 (ref. ^21^), C102 (ref. ^22^), Fab2-36 (ref. ^23^) and P2B-2F6 (ref. ^24^) were produced in 293-6E cells (National Research Council of Canada) by co-transfection of heavy and light chain expression plasmids using 25 kDa branched polyethylenimine (Sigma-Aldrich, cat. no. 408727). Following 6–7 days of incubation at 37 °C and 6% CO_2_ under constant shaking at 110 r.p.m. in FreeStyle Expression Medium (Thermo Fisher, cat. no. 12338001) supplemented with 0.2% penicillin/streptomycin (Thermo Fisher, cat. 15140122), culture supernatants were collected, clarified by centrifugation and filtration (0.45 µm), and incubated with Protein G Sepharose 4 FastFlow (GE Life Sciences, cat. no. 17061805). Subsequently, antibodies were eluted from Protein G beads using 0.1 M glycine (pH 3.0) in chromatography columns (Bio-Rad) and buffered in 1 M Tris (pH 8.0). Final buffer exchange to PBS was performed using Amicon spin membranes (Millipore). Antibodies REGN10933 and REGN10987 (ref. ^25^) were produced and kindly provided by Boehringer Ingelheim. Antibodies etesevimab (also known as CB6, ref. ^26^), bamlanivimab (also known as LY-CoV555, ref. ^27^), and DZIF-10c (derived from antibody HbnC3t1p1_F4, refs. ^28,29^) were obtained from stocks of clinical product.

### Pseudovirus neutralization assays

Pseudovirus neutralization assays were performed using a single-round infection lentivirus-based system^10,30^. Pseudovirus particles were generated in HEK293T cells by co-transfection of plasmids encoding for the SARS-CoV-2 spike protein, HIV-1 Tat, HIV-1 Gag/Pol, HIV-1 Rev, and luciferase followed by an internal ribosome entry site (IRES) and ZsGreen using FuGENE 6 Transfection Reagent (Promega, cat. no. E2691). Culture supernatants were changed to fresh medium 24 h after transfection, and pseudovirus-containing supernatants were collected at 48–72 h after transfection. Following centrifugation and filtration (0.45 µm), pseudoviruses were stored at −80 °C until use. Pseudoviruses were titrated by infecting 293T-ACE2 cells, and the luciferase activity was assessed, following a 48 h incubation period at 37 °C and 5% CO_2_, by addition of luciferin/lysis buffer (10 mM MgCl_2_ (cat. no. M8266), 0.3 mM ATP (cat. no. A26209), 0.5 mM Coenzyme A (cat. no. C3144), 17 mM IGEPAL (cat. no. I8896) (all Sigma-Aldrich), and 1 mM D-Luciferin (GoldBio, cat. no. LUCNA-1G) in Tris-HCL) using a microplate reader (Berthold). Pseudovirus dilutions resulting in an at least 1,000-fold difference in relative light units (RLUs) between infected and non-infected 293T-ACE2 cells were used for neutralization assays.

Serum samples were collected by centrifugation and stored at −80 °C until analysis. Serum samples were heat-inactivated at 56 °C for 45 min prior to use. Serial dilutions of serum (1:3 dilution series with a starting dilution of 1:10) and monoclonal antibodies (1:5 dilution series with a starting concentration of 10 µg ml^−1^) were co-incubated with pseudovirus supernatants for 1 h at 37 °C prior to the addition of 293T-ACE2 cells. Following a 48 h incubation at 37 °C and 5% CO_2_, luciferase activity was determined as described above. Serum samples were tested in single dilution series and monoclonal antibodies were tested in duplicates. Background RLUs of non-infected cells were subtracted, and the 50% inhibitory serum dilution (ID_50_) and 50% inhibitory concentration of monoclonal antibodies were determined as the serum dilution and antibody concentration, respectively, resulting in a 50% RLU reduction compared with virus-infected untreated control cells. This was assessed using a non-linear fit model to plot an agonist versus normalized dose response curve with variable slope using the least squares fitting method in GraphPad Prism 7.0 (GraphPad).

### Statistical methods

Comparison of neutralizing titers between different variants was performed using a two-tailed Wilcoxon matched-pairs signed rank test. Serum samples that did not achieve 50% inhibition at the lowest tested dilution of 10 (lower limit of quantification, LLOQ) were imputed to half of the LLOQ (ID_50_ = 5) for graphical representation and statistical evaluation.

### Reporting Summary

Further information on research design is available in the Nature Research Reporting Summary linked to this article.

## Online content

Any methods, additional references, Nature Research reporting summaries, source data, extended data, supplementary information, acknowledgements, peer review information; details of author contributions and competing interests; and statements of data and code availability are available at 10.1038/s41591-021-01676-0.

## Supplementary information

Supplementary Information Supplementary Tables 1 and 2.

Reporting Summary

## Data Availability

All data are included in the figures and supplementary information. Requests for additional data should be directed to the corresponding author and may be subject to restrictions based on data and privacy protection regulations and/or require a Material Transfer Agreement (MTA).
