# Supplementary material for: mRNA booster immunization elicits potent neutralizing serum activity against the SARS-CoV-2 Omicron variant
Source: Nat Med. 2022 Jan 19;28(3):477–80. doi: 10.1038/s41591-021-01676-0 (PMC8767537; doi:10.1038/s41591-021-01676-0)
Supplement: Supplementary file 1 — Supplementary Tables 1 and 2. [file 41591_2021_1676_MOESM1_ESM.pdf]

nature portfolio

https://doi.org/10.1038/s41591-021-01676-0

Supplementary information

mRNA booster immunization elicits potent neutralizing serum activity against the SARS-CoV-2 Omicron variant

In the format provided by the authors and unedited

Supplementary Table 1. *Study participant demographics.*

a

**Vaccinated cohort**	
**Participants** - *n*	30
**Gender**	
Male - *n* (%)	13 (43%)
Female - *n* (%)	17 (57%)
**Age** - median years (range)	49 (27-78)
**Reported comorbidities**	
Cardiovascular disease - *n* (%)	10 (33.3%)
Respiratory disease - *n* (%)	4 (13.3%)
Rheumatoid arthritis - *n* (%)	2 (6.6%)
Diabetes - *n* (%)	1 (3.3%)
Polymyalgia rheumatica - *n* (%)	1 (3.3%)
Autoimmune hepatitis - *n* (%)	1 (3.3%)
**Body mass index** - median (IQR, range)*	26.0 (24.2-29.6; 18.8-37.2)
**Vaccination received**	BNT162b2
**Sampling time point -** median weeks (IQR; range)	
Early (after second dose)	3.9 (3.7-4.2; 2.9-6.0)
Late (after second dose)	21.0 (20.3-22.9; 18.7-31.1)
Booster (after second dose)	40.6 (36.8-41.8; 29.7-44.0)
**Time between second and third dose** - median weeks (IQR; range)	36.6 (32.2-38.5; 26.9-40.9)

* available for 29 participants.

b

**Convalescent cohort**	
**Participants** - *n*	30
**Gender**	
Male - *n* (%)	14 (47%)
Female - *n* (%)	16 (53%)
**Age** - median years (range)	52 (22-68)
**Reported comorbidities**	
Arterial hypertension - *n* (%)	3 (10%)
Tumor - *n* (%)	3 (10%)
Asthma - *n* (%)	3 (10%)
Diabetes - *n* (%)	1 (3.3%)
Multiple sclerosis - *n* (%)	1 (3.3%)
Coagulation disorder - *n* (%)	1 (3.3%)
**Time period of SARS-CoV-2 infection**	February - March 2020
**COVID-19 severity**	
Mild symptoms - *n (%)*	29 (97%)
Hospitalized - n (%)	1 (3%)
**Vaccination received**	BNT162b2
**Sampling time point** - median weeks (IQR; range)	
Early (after disease onset)	6.0 (4.7-7.0; 3.6-10)
Late (after disease onset)	52.9 (51.4-56.8; 49.4-59.4)
Hybrid (after disease onset)	66.8 (65.3-68.4; 62.4-71.4)
**Time between infection and vaccination** - median weeks (IQR; range)	60.9 (58.9-61.8; 52.4-66.1)

Supplementary Table 2. *Serum neutralizing activity against Wu01 and Omicron.*

Vaccinated cohort

Study ID	Wu01 Serum ID_{50}			Omicron Serum ID_{50}		
	Early	Late	Boost	Early	Late	Boost
Pt. #001	41	<10	5,079	<10	<10	324
Pt. #002	37	14	2,537	<10	<10	624
Pt. #003	293	28	2,545	<10	<10	516
Pt. #004	242	75	1,849	<10	<10	2,410
Pt. #005	264	41	7,706	<10	<10	2,907
Pt. #006	1,749	339	11,807	12	<10	2,872
Pt. #007	689	231	6,149	<10	11	1,485
Pt. #008	588	267	9,734	<10	<10	1,690
Pt. #009	254	115	2,948	<10	<10	756
Pt. #010	1,478	467	22,912	28	<10	881
Pt. #011	1,342	372	22,833	33	37	5,510
Pt. #012	726	94	6,211	<10	<10	1,148
Pt. #013	963	430	6,909	35	44	1,741
Pt. #014	3,336	817	11,718	114	21	3,050
Pt. #015	1,006	165	5,567	15	<10	428
Pt. #016	521	101	10,154	<10	<10	2,484
Pt. #017	823	54	3,077	<10	<10	1,185
Pt. #018	1,128	265	8,731	<10	11	924
Pt. #019	1,008	239	6,461	<10	<10	601
Pt. #020	1,334	225	6,269	<10	14	450
Pt. #021	695	535	11,128	<10	36	2,645
Pt. #022	1,600	326	19,819	49	134	7,851
Pt. #023	294	227	7,643	<10	<10	868
Pt. #024	311	98	11,463	23	53	3,514
Pt. #025	474	160	3,768	<10	<10	1,312
Pt. #026	551	327	6,078	<10	<10	1,571
Pt. #027	831	112	6,317	10	<10	625
Pt. #028	408	118	7,307	<10	23	1,454
Pt. #029	270	93	3,676	<10	21	2,239
Pt. #030	352	68	609	<10	<10	21

Convalescent cohort

Study ID	Wu01 Serum ID_{50}			Omicron Serum ID_{50}		
	Early	Late	Hybrid	Early	Late	Hybrid
R014	330	<10	5,178	<10	<10	141
R047	2,927	566	8,932	<10	<10	1,657
R056	1,355	88	4,137	<10	<10	74
R082	3,144	108	7,948	<10	<10	2,188
R090	623	119	8,650	<10	<10	4,528
R102	862	90	27,730	<10	<10	3,187
R137	204	141	8,581	<10	<10	430
R164	669	70	5,818	<10	<10	725
R212	217	80	5,347	<10	<10	343
R238	731	140	14,687	87	76	11,001
R244	2,505	119	24,552	<10	<10	5,843
R247	1,032	142	13,058	<10	14	2,034
R256	8,735	397	23,738	<10	31	16,570
R257	394	353	9,332	<10	20	907
R285	264	75	3,450	<10	<10	3,888
R289	217	153	7,964	<10	<10	1,676
R297	547	89	30,227	<10	<10	3,397
R299	73	30	5,709	<10	<10	1,233
R317	438	133	9,163	<10	<10	2,205
R399	76	26	2,338	<10	<10	474
R401	59	38	4,610	<10	<10	1,278
R434	473	137	5,264	<10	<10	430
R440	1,250	381	21,847	<10	11	1,375
R458	171	14	2,553	<10	<10	874
R484*	971	289	15,642	17	34	8,297
R491	421	99	7,680	<10	17	806
R527	37	18	2,979	<10	<10	400
R715	91	55	6,999	<10	<10	2,381
R749	11,008	95	4,579	79	31	8,028
R849	219	160	7,640	<10	11	4,546

Serum ID_{50}				
>2,500	250-2,500	50-250	10-50	<10

* Participant R484 received two BNT162b2 vaccinations three weeks apart.
