# Supplementary material for: mRNA booster immunization elicits potent neutralizing serum activity against the SARS-CoV-2 Omicron variant
Source: Nat Med. 2022 Jan 19;28(3):477–80. doi: 10.1038/s41591-021-01676-0 (PMC8767537; doi:10.1038/s41591-021-01676-0)
Supplement: Supplementary file 2 — Reporting Summary [file 41591_2021_1676_MOESM2_ESM.pdf]

nature portfolio

Corresponding author(s): Florian Klein

Last updated by author(s): Dec 21, 2021

Reporting Summary

Nature Portfolio wishes to improve the reproducibility of the work that we publish. This form provides structure for consistency and transparency in reporting. For further information on Nature Portfolio policies, see our Editorial Policies and the Editorial Policy Checklist.

Statistics

For all statistical analyses, confirm that the following items are present in the figure legend, table legend, main text, or Methods section.

n/a	Confirmed	
☐	☒	The exact sample size (*n*) for each experimental group/condition, given as a discrete number and unit of measurement
☐	☒	A statement on whether measurements were taken from distinct samples or whether the same sample was measured repeatedly
☐	☒	The statistical test(s) used AND whether they are one- or two-sided<br>*Only common tests should be described solely by name; describe more complex techniques in the Methods section.*
☒	☐	A description of all covariates tested
☒	☐	A description of any assumptions or corrections, such as tests of normality and adjustment for multiple comparisons
☐	☒	A full description of the statistical parameters including central tendency (e.g. means) or other basic estimates (e.g. regression coefficient) AND variation (e.g. standard deviation) or associated estimates of uncertainty (e.g. confidence intervals)
☒	☐	For null hypothesis testing, the test statistic (e.g. *F*, *t*, *r*) with confidence intervals, effect sizes, degrees of freedom and *P* value noted<br>*Give P values as exact values whenever suitable.*
☒	☐	For Bayesian analysis, information on the choice of priors and Markov chain Monte Carlo settings
☒	☐	For hierarchical and complex designs, identification of the appropriate level for tests and full reporting of outcomes
☒	☐	Estimates of effect sizes (e.g. Cohen's *d*, Pearson's *r*), indicating how they were calculated

Our web collection on statistics for biologists contains articles on many of the points above.

Software and code

Policy information about availability of computer code

Data collection	No code was used.
Data analysis	Neutralization data was analyzed using GraphPad Prism version 7.0

For manuscripts utilizing custom algorithms or software that are central to the research but not yet described in published literature, software must be made available to editors and reviewers. We strongly encourage code deposition in a community repository (e.g. GitHub). See the Nature Portfolio guidelines for submitting code & software for further information.

Data

Policy information about availability of data

All manuscripts must include a data availability statement. This statement should provide the following information, where applicable:

- Accession codes, unique identifiers, or web links for publicly available datasets
- A description of any restrictions on data availability
- For clinical datasets or third party data, please ensure that the statement adheres to our policy

All data are included in the figure, table, or supplement of this manuscript.

Field-specific reporting

Please select the one below that is the best fit for your research. If you are not sure, read the appropriate sections before making your selection.

☒ Life sciences ☐ Behavioural & social sciences ☐ Ecological, evolutionary & environmental sciences

For a reference copy of the document with all sections, see nature.com/documents/nr-reporting-summary-flat.pdf

Life sciences study design

All studies must disclose on these points even when the disclosure is negative.

Sample size: No formal sample size calculation was performed. 30 participants from the convalescent and vaccinated cohorts were selected based on sample availability and similar distributions of age and gender.

Data exclusions: No data was excluded from analysis. Serum samples that did not achieve 50% inhibition at the lowest tested dilution of 10 (lower limit of quantification, LLOQ) were imputed to ½ of the LLOQ (ID50=5) for graphical representation and statistical evaluation.

Replication: Each serum sample was tested once against each analyzed pseudovirus strain in a single dilution series. Monoclonal antibodies were tested in technical duplicates against each pseudovirus strain and the average IC50 values are reported.

Randomization: Serum samples were obtained as part of longitudinal observational cohorts assessing the immune response after convalescence or after vaccination provided as routine care. Any vaccination was provided outside of the study protocols, so randomization was not applicable.

Blinding: Participants were enrolled into open-label observational cohorts. Vaccinations were not provided as part of the study protocols but as routine care. As a consequence, neither participants nor investigators were blinded to the receipt of vaccinations.

Reporting for specific materials, systems and methods

We require information from authors about some types of materials, experimental systems and methods used in many studies. Here, indicate whether each material, system or method listed is relevant to your study. If you are not sure if a list item applies to your research, read the appropriate section before selecting a response.

Materials & experimental systems

n/a	Involved in the study	
☐	☒	Antibodies
☐	☒	Eukaryotic cell lines
☒	☐	Palaeontology and archaeology
☒	☐	Animals and other organisms
☐	☒	Human research participants
☒	☐	Clinical data
☒	☐	Dual use research of concern

Methods

n/a	Involved in the study	
☒	☐	ChIP-seq
☒	☐	Flow cytometry
☒	☐	MRI-based neuroimaging

Antibodies

Antibodies used:
Bamlanvimab (Jones et al.; PDB entry 7KMG)
Etesevimab (Shi et al., PDB entry 7C01)
REGN10933 (casirivimab) (Hanssen et al., PDB entry 6XDG)
REGN10987 (imdevimab) (Hanssen et al., PDB entry 6XDG)
S309 (sotrovimab) (Pinto et al., PDB entry 6WPS)
C102 (Robbiani et al., PDB entry 7K8M)
Fab2-36 (Liu et al., PDB entry 7N5H)
DZIF-10c (Kreer et al.; Halwe et al.; GenBank IDs QKY76686.1 and QKY76714.1)
P2B-2F6 (Bin Ju et al., PDB entry 7BWJ)

Validation:
Plasmids for antibody production were verified by sequencing.
For Bamlanvimab, Etesevimab, and DZIF-10c, batches produced for clinical application were used.

Eukaryotic cell lines

Policy information about cell lines

Cell line source(s):
HEK293T cells (ATCC Catalog# CRL-11268)
293T-ACE2 cells (BEI resources Catalog # 52511)

	293-6E cells (NRC file 11565)
Authentication	Cell lines were not authenticated.
Mycoplasma contamination	Cell lines were not checked for Mycoplasma contamination.
Commonly misidentified lines (See ICLAC register)	No commonly misidentified cell lines were used in this study.

Human research participants

Policy information about studies involving human research participants

Population characteristics	COVID-19 convalescent cohort:<br>30 convalescent individuals, 14 males and 16 females, with a median age of 52 years. Individuals were SARS-CoV-2-infected in February or March 2020. 29/30 convalescent individuals received one dose of BNT162b2 at a median of 15 months after disease onset. One individual received an additional dose of BNT162b2 at 3 weeks after the first dose.<br>Vaccinated cohort:<br>30 vaccinated individuals, 13 males and 17 females, with a median age of 49 years. All individuals received 2 doses of BNT162b2, approximately three weeks apart followed by a third dose at a median of 8.5 months after the second dose.
Recruitment	COVID-19 convalescent cohort:<br>Participants were invited to participate in the cohort when fulfilling the inclusion criteria of i.) age ≥18 years, ii.) history of SARS-CoV-2 positive polymerase chain reaction (PCR) from a respiratory swab or collected sputum, and/or iii.) an onset of COVID-19 specific symptoms more than 3 weeks ago. Participants enrolled in this cohort were followed longitudinally in an outpatient study clinic to assess the longitudinal changes in the immune response to SARS-CoV-2 after vaccination. Participants received financial compensation and were informed about their antibody levels to SARS-CoV-2, when assessed. It is possible that individuals with long-term symptoms would show higher retention rates, but this would not affect immunogencity results and the conclusions drawn.<br>Vaccinated cohort:<br>Samples were obtained from two longitudinal cohorts of vaccinated individuals that analyze the long-term immune response to COVID-19 vaccines. The cohorts were composed of vaccinated health care workers that were offered participation, as well as of elderly individuals (70 years and higher) of a general practice that were offered participation irrespective of medical conditions. Participants underwent NAAT at times of sampling. No samples from individuals with positive SARS-CoV-2 testing by NAAT or anti-nucleocapsid serology or history of SARS-CoV-2 infection were considered for analysis in this study. Participants received financial compensation for their participation.<br><br>All participants in all cohorts provided written informed consent for their participation. Neither initial vaccinations nor booster immunizations were offered within the observational studies, and all vaccinations were performed within routine care. Samples and participants for analysis were selected post-hoc (i.e., after booster immunizations had been performed within routine care) and chosen to be overall comparable (administered vaccines, temporal relationship of sampling visits to vaccinations, sex and age distribution). Overall, participants were relatively healthy which may be associated with a higher response to vaccinations compared to less healthy cohorts.
Ethics oversight	COVID-19-convalescent samples were obtained under protocols approved by the ethics committee (EC) of the Medical Faculty of the University of Cologne (16-054 and 20-1187).<br>Samples from non-infected vaccinated individuals were obtained under protocols (EICOV, COVIMMUNIZE, and COVIM) approved by the EC of Charité - Universitätsmedizin Berlin (EA4/245/20 and EA4/244/20; EICOV and COVIMMUNIZE), and by the Federal Institute for Vaccines and Biomedicines (Paul Ehrlich Institute) and the EC of the state of Berlin (COVIM).

Note that full information on the approval of the study protocol must also be provided in the manuscript.
